# Improvement in Midline Shift Is a Positive Prognostic Predictor for Malignant Middle Cerebral Artery Infarction Patients Undergoing Decompressive Craniectomy

**DOI:** 10.3389/fneur.2021.652827

**Published:** 2021-05-20

**Authors:** Xin Chen, Qiang Hao, Shu-Zhe Yang, Shuo Wang, Yuan-Li Zhao, Dong Zhang, Xun Ye, Hao Wang

**Affiliations:** Department of Neurosurgery, Beijing Tiantan Hospital, Capital Medical University, Beijing, China

**Keywords:** malignant middle cerebral artery infarction, decompressive craniectomy, mortality, hypertensive intracerebral hemorrhage, improvement in midline shift

## Abstract

**Objective:** The aim of this retrospective study is to evaluate the risk factors of malignant middle cerebral artery infarction (MMCAI) patients and explore an applicable prognostic predictor for MMCAI patients undergoing decompressive craniectomy (DC).

**Methods:** Clinical data from the period 2012–2017 were retrospectively evaluated. Forty-three consecutive MMCAI patients undergoing DC were enrolled in this study. The 30-day mortality was assessed, and age, location, hypertension, pupil dilation, onset to operation duration, midline shift, and Glasgow Coma Scale (GCS) score were identified by univariate analysis and binary logistic regression.

**Results:** In this retrospective study for DC patients, the 30-day mortality was 44.2%. In the univariate analysis, advanced age (≥60 years), right hemispheric location, hypertension, pupil dilation, shorter onset to operation duration (<48 h), improved midline shift (*t* = 4.214, *p* < 0.01), and lower pre-operation GCS score were significant predictors of death within 30 days. In binary logistic regression analysis, age [odds ratio (OR) = 1.141, 95% CI 1.011–1.287], the improvement of the midline shift (OR = 0.764, 95% CI 0.59–0.988), and pupillary dilation (OR = 15.10, 95% CI 1.374–165.954) were independent influencing factors. For the receiver operating characteristic (ROC) analysis of the relationship between post-operation outcomes and midline shift improvement, the area under the curve (AUC) was 0.844, and the cutoff point of midline shift improvement was 0.83 cm.

**Conclusion:** Improved midline shift was a significant predictor of 30-day mortality. The improved midline shift of >0.83 cm indicated survival at 30 days.

## Introduction

Malignant middle cerebral artery infarction (MMCAI) is a kind of large hemispheric infarction because of the occlusion of the proximal middle cerebral artery or the internal carotid artery. MMCAI is the most critical and severe form of acute stroke with mortality up to 80% (1). Acute management should include rapid recanalization by intravenous/intra-arterial (IA) thrombolysis and IA mechanical thrombectomy in the time window for restoration of cerebral blood flow (2). However, if it is beyond the therapeutic time window, MMCAI can cause acute and life-threatening brain swelling due to the post-ischemic edema. In many cases, even with the intravenous/IA thrombolysis and IA mechanical thrombectomy, the edema is inescapable. The post-ischemic edema is the main cause of death and severe complications because malignant edema could lead to compression of the brain stem, occlusive hydrocephalus, and secondary ischemic damage presenting with clinical deterioration, consciousness declining, herniation, and death within 2–5 days (3).

Among the limited number of options for MMCAI, decompressive craniectomy (DC) has been proven to be an effective way to reduce mortality, which could allow immediate decompression of the brain and the release of the intracranial hypertension (4–6). The procedure is *via* an ipsilateral frontoparietotemporal craniotomy, followed by plastic reconstruction of the dura mater. Some clinical trials have clearly demonstrated that DC significantly increases the survival probability of MMCAI patients (7, 8).

Patient selection for DC relies on baseline patient characteristics, neurological presentation, imaging evaluation (such as midline shift), and time from the onset of symptoms to surgery (9, 10). The correlation of specific clinical variables to patient mortality helps inform surgeons and families about the patient's potential prognosis.

In the study, we analyzed the 30-day outcome in a cohort of consecutive patients undergoing DC and analyzed patient characteristics and clinical variables for predictors of survival to identify subpopulations that benefit most from surgical intervention.

## Methods

### Study Design and Population

We included in this study all 43 identified patients who were diagnosed with middle cerebral artery (MCA) stroke and underwent DC for malignant MCA syndrome during the time period of July 2012 to October 2017. Information on the following data was collected: age, gender, infarction sides, infarction type, pupillary dilation before operation, onset to operation duration, intra-operation bleeding, duration of operation, pre-operation Glasgow Coma Scale (GCS) score; past medical history; improvement of the midline shift after DC; and mortality at 30 days. The midline shifting distance was the distance between the benchmark and the septum pellucidum that deviated the farthest from it. Improvement in midline shift is defined as the difference between pre-DC and post-DC midline shifting distances (pre-DC values minus post-DC values). So the positive values mean that the septum pellucidum shifted back to the benchmark after DC. GCS score and pupillary response were documented for malignant MCA infarction at the time of clinical deterioration prior to surgery. The infarction type is divided into arterial thrombotic cerebral infarction (ATCI), atherosclerotic cerebral infarction (ACCE), and undifferentiated type of infarction (UT). All the enrolled patients are right-handed. To evaluate outcomes, the status at 30 days after DC was documented as survival or not. All measurements were performed by two neurosurgeons (X.C. and H.W.) blinded to all other data of the patient at the time the measurements were taken.

DC surgery was performed according to a standardized operative procedure that is based on a frontoparietotemporal craniotomy, ipsilateral to the lesion, followed by plastic reconstruction of the dura mater, allowing immediate decompression of the brain.

A craniectomy was performed in patients who developed clinical deterioration signs including pupil asymmetry, altered mental status, and progressive hemiplegia. The study protocol was approved by the institutional review board of Beijing Tiantan Hospital, Capital Medical University. Informed consent was not obtained because of the retrospective nature of the study.

### Statistical Analysis

Categorical variables are expressed as number (%) and continuous variables as the mean ± standard deviation when the data followed a normal distribution. Categorical variables were compared using the chi-square test or Fisher's exact probability test, and the medians were compared using the Student's *t*-test. Chi-square test and Fisher's exact test were used to test the relationships between continuous variables and postoperative outcomes. Variables with *p*-values < 0.05 on univariate analysis were brought into the binary logistic regression to determine independent risk factors. Odds ratios (ORs) and 95% confidence intervals (CIs) were calculated. For the variable of the midline shift, the receiver operating characteristic (ROC) curve analysis was applied to determine the predictive value and cutoff point. All statistical tests were two-sided, and the level of significance was set at *p* < 0.05. All statistical analyses were performed using the Statistical Package for the Social Sciences software version 22 (SPSS, Chicago, IL, USA).

## Results

### Patient Characteristics and Clinical Variables

The patient characteristics and clinical variables are summarized in Table 1. Forty-three consecutive patients (32 males vs. 11 females) who presented with MMCAI and underwent DC were enrolled in this study. The mean age was 53 ± 10 years, ranging from 24 to 77 years, 32 (74%). Twenty-one patients had infarction involving the left hemisphere, and 22 patients had a lesion in the right hemisphere. Hypertension was diagnosed in 24 patients, heart disease in 16, and diabetes in 10. The mean pre-operation GCS score was 7 ± 3, ranging from 3 to 13. The average intra-operation bleeding was 281.40 ± 321.27 ml, ranging from 50 to 2,000 ml. The mean time of DC operation was 112.51 ± 28.98 min. Before the DC operation, 13 patients had pupillary dilation. After the operation, the midline had a mean return distance of 10.12 ± 0.92 mm (-11.30–10.12 mm).

**Table 1:** Univariate analysis shows the relationships between variables and 30-day mortality.

**Parameters**	**Parametric subclass**	**Parametric description**		**Chi-square test (*X*^**2**^)/Fisher exact test/*t*-test (*t*)**	P*
		**Survivors**	**Non-survivors**		
Gender	Male	19	13	-	0.495
	Female	5	6		
Age	≥60	3	8	χ^2^ = 4.882	0.027*
	<60	21	11		
Location	Right	9	13	χ^2^ = 5.721	0.017*
	Left	15	6		
Infarction type	UT	2	0	χ^2^ = 0.1667	0.435
	ATCI	13	11		
	ACCE	9	8		
Hypertension	Y	10	14	χ^2^ = 4.408	0.036*
	N	14	5		
Heart disease	Y	8	8	χ^2^ = 0.349	0.555
	N	16	11		
Diabetes	Y	4	6	-	0.295
	N	20	13		
Pupillary dilation	Y	2	11	χ^2^ = 12.35	<0.01*
	N	22	8		
Pre-op GCS score	-	7.75 ± 2.09	6.16 ± 2.79	*t* = 2.138	0.038*
Onset to op duration	≥48	19	9	χ^2^ = 4.721	0.030*
	<48	5	10		
Intra-op bleeding	-	327.08 ± 410.46	223.68 ± 138.81	*t* = 1.049	0.3
Duration of operation	-	115.42 ± 27.07	108.84 ± 31.59	*t* = 0.735	0.467
Midline shift improvement	-	3.24 ± 3.21	−2.03 ± 4.78	*t* = 4.214	<0.01*

*Op, operation; GCS, Glasgow coma scale; UT, undifferentiated type of infarction; ATCI, arterial thrombotic cerebral infarction; ACCE, atherosclerotic cerebral infarction*.

### Predictors of Death at 30 Days

Patient characteristics and clinical variables were investigated in relation to 30-day mortality to identify potential predictors.

**Figure 1:**
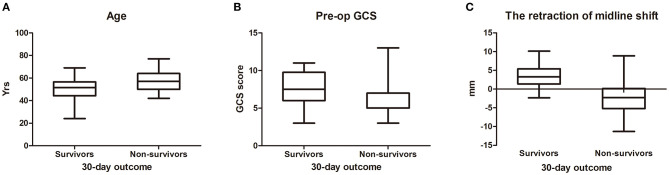
The difference between survivors and non-survivors for measurement data (30-day outcome). The distribution of age **(A)**, pre-op GCS **(B)**, and the retraction of midline shift **(C)** between survivors and non-survivors. For **(C)**, the negative reading means worsening of midline shift after DC.

**Figure 2:**
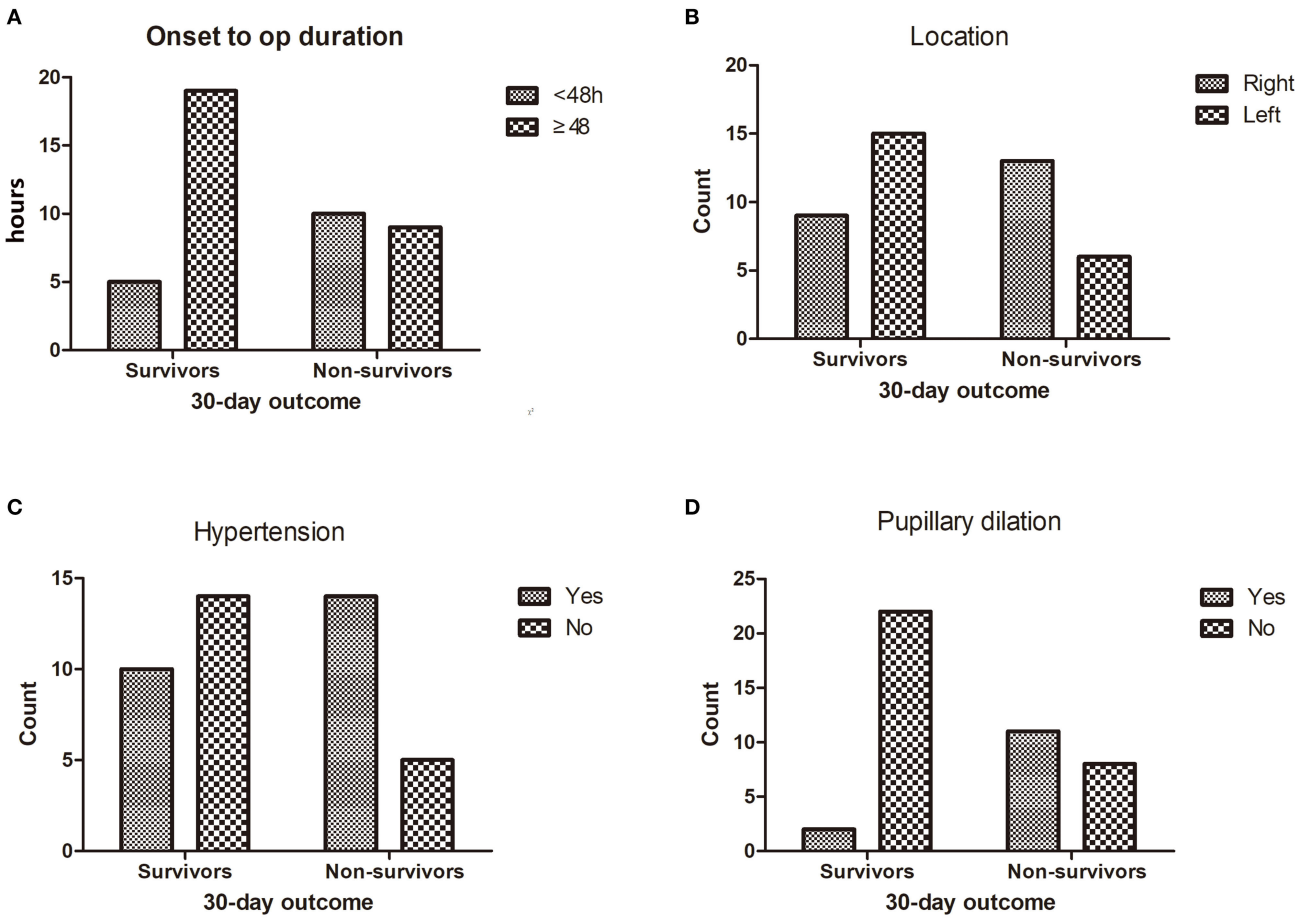
The difference between survivors and non-survivors for enumeration data (30-day outcome).The distribution of onset to op duration **(A)**, location **(B)**, hypertension **(C)** and pupillary dilation **(D)** between survivors and non-survivors.

In univariate analysis (Table 1), age ≥60 (χ^2^ = 4.882, *p* = 0.027) (Figure 1A), shorter onset to operation duration (< 48 h) (Figure 2A) (χ^2^ = 4.721, *p* = 0.030), right hemispheric location (χ^2^ = 5.721, *p* = 0.027) (Figure 2B), a history of hypertension (χ^2^ = 4.408, *p* = 0.036) (Figure 2C), pupillary dilation (χ^2^ = 12.35, *p* < 0.01) (Figure 2D), higher pre-operation GCS (*t* = 2.138, *p* = 0.038) (Figure 1B), and lower midline shift improvement (*t* = 4.214, *p* < 0.01) (Figure 1C) were significantly more likely to have a poor outcome of death at 30 days post-DC.

In the multivariable analysis (Table 2), patients with increasing age (OR = 1.141, 95% CI 1.011–1.287) and pupillary dilation (OR = 15.10, 95% CI 1.374–165.954) were more likely to have a poor outcome of death after DC, while the midline shift improvement (OR = 0.764, 95% CI 0.59–0.988) was a protective factor against the poor ending of death.

**Table 2:** Binary logistic regression shows the independent factors of postoperative outcomes.

**Parameters**	***P***	**OR**	**OR 95% C.I**.
Age	0.032	1.141	1.011–1.287
The retraction of midline shift	0.04	0.764	0.59–0.988
Pupillary dilation	0.026	15.099	1.374–165.954

**Figure 3:**
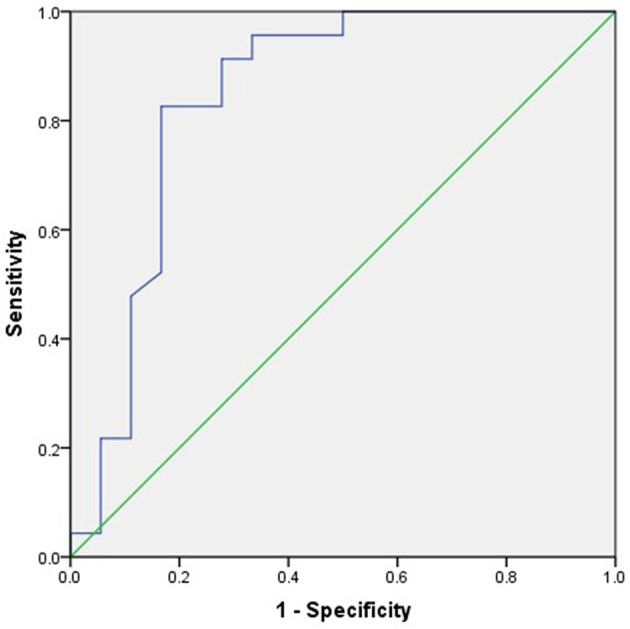
The receiver operating characteristic (ROC) analysis of the relationship between 6-month outcome and midline shift improvement. The area under the curve (AUC) was 0.844, and the cutoff point of midline shift retraction was 0.83 cm.

For the ROC analysis of the relationship between post-operation outcomes and midline shift improvement, the area under the curve (AUC) was 0.844 and the cutoff point of midline shift improvement was 0.83 cm (Figure 3).

## Discussion

DC as a lifesaving procedure that is often performed in patients with space-occupying lesions of various underlying pathologies, especially during the treatment of space-occupying ischemic stroke (11–14). We retrospectively reviewed the 30-day clinical outcome in patients undergoing DC by analyzing a 5-year, single-center, consecutive patient cohort. Overall, advanced age, right hemispheric location, hypertension, pupil dilation, improved midline shift, and lower GCS score were significant predictors of death.

In this retrospective study for DC patients, the 30-day mortality was 44.2%. It has been reported in other studies that mortality of MMCAI approaches 80% without DC surgery. We show that DC could reduce mortality as other researchers did (14, 15). DC relieves the constraint of bulgy intracranial contents and protects the important structures in the brain stem from compression.

Several studies have reported that age should be considered as an important factor in patients who undergo surgical decompression (16–19). We showed that age ≥60 years was an independent factor for the prognosis of patients with DC (Tables 1, 2, Figure 1A). The mortality of DC patients in the age ≥60 group was higher than that of patients at a younger age (χ^2^ = 4.882, *p* = 0.027). The aging caused physical and functional deterioration, with declining flexibility and immunity. So, the older patients found it harder to recover from the MMCAI and surgical attack than the younger patients.

Previous studies have recommended performing DC for the treatment of malignant cerebral infarction in patients before clinical or radiological signs of brain herniation, within a time frame of 48 h, but in this series, we obtained the opposite result that after more than 48 h of onset to operation, duration was a significant positive predictor for positive patient outcomes. The average time of onset to operation in this series was 79.22 ± 76.26 h. Early DC (<48 h) did not show superiority over a late one (Table 1, Figure 2A). It may be because that all enrolled early DC patients were more critical than the late DC patients. In this series, most enrolled patients were transferred from the neurology department or intensive care unit. The process of consultation, benefit–risk balance between family members, and transfer also cost extra time and made the onset to operation period more than 48 h. Otherwise, most patients were implemented a therapeutic regimen of intense dehydration (megadose of mannitol, i.e., 20% mannitol, q8h). The dehydration management maintained the relative steady status and prevented clinical deterioration until loss of balance. However, by now, there is no convincing study to show an optimal time frame for DC. In the three most authoritative trials [Decompressive Surgery for the Treatment of Malignant Infarction of the Middle Cerebral Artery (DESTINY), Decompressive Craniectomy in Malignant Middle Cerebral Artery Infarction (DECIMAL), and Hemicraniectomy After Middle Cerebral Artery Infarction With Life-threatening Edema Trial (HAMLET)], 48 h was adopted as a constant for the DC, and surgical procedures showed an advantage over medical management at reducing mortality. According to the clinical experience in our center, early DC is indispensable, especially when the clinical status deteriorated.

In this series, mortality of patients with MMCAI at the side of the non-dominant hemisphere (right) was 59.10%, higher than that (28.57%) of the dominant hemisphere (left) (χ^2^ = 5.721, *p* = 0.017) (Table 1, Figure 2B). It is probably because the sequelae of infarct does not appear until the patients become symptomatic. For most patients, the left hemisphere is dominant with the most important linguistic function. So, left-sided strokes are more symptomatic and are reported earlier. Whereas, right-sided strokes are more occult and not brought to light until it becomes apparently symptomatic, therefore having a longer onset-to-thrombectomy duration. In a previous study concerning patient outcome after DC with severe ischemic stroke, there were no differences between left- and right-sided infarctions about the functional outcome (20). Park et al. (21) concluded that no significant difference in the clinical outcome with modified Rankin Scale (mRS) scores was observed between surviving patients with dominant and non-dominant hemisphere infarction. While in another research, patients with cerebral infarction involving the dominant hemisphere had higher odds of unfavorable functional outcome at 90 days than their counterparts (4). The functional anomaly of the dominant hemisphere could lead to language dysfunction and lower the functional outcome. However, the link between MMCAI of the non-dominant hemisphere (right) and higher mortality is unknown.

Hypertension destroys the structure and function of the circulatory system and further damages vital organs (such as the brain and the heart) and increases bleeding in operations. In a previous study (22), hypertension was not related to the 30-day mortality of patients with DC for MMCAI. Nevertheless, in this series, hypertension was a significant contributor to bad outcomes. The mortality of patients with hypertension (58.33%) was higher than those without hypertension (26.32%) (Table 1, Figure 2C). So, it is important to control blood pressure during the perioperative period for MMCAI patients.

GCS score is a reliable evaluation method for the coma degree. Pre-operation GCS score represents a comprehensive status of consciousness, physical activity, and language. Goedemans et al. (16) reported that poor GCS score was a significant predictor of unfavorable outcome for patients after DC. In our study, lower pre-operation GCS score was significantly related to 30-day mortality.

Worsening clinical status was defined as pupillary dilation and signs of cerebral herniation or severe brain edema with mass effect despite medical treatment or hemorrhagic infarction. When a bright light gets into the eyes, the iris sphincter muscles contract to make the pupil shrink and protect the contents in the eyes. The process is controlled by the brain stem. When the brain stem shifts, the pupil reflex disappears and causes pupillary dilation. Patients with pupillary abnormalities were significantly more likely to have poor outcomes (4, 16, 18, 23). In our series, pupillary dilation was strongly linked to 30-day mortality (χ^2^ = 12.35, *p* < 0.01) (Table 1, Figure 2D). Multivariable analysis also showed that pupillary dilation was an independent predictor for 30-day mortality (OR = 15.099, 95% CI 1.374–165.954) (Table 2). So, we recommend that early and meticulous pupillary checks are important for MMCAI patients before the pupils amplify.

The midline shift is an important indication for DC. In a previous study, Sang-Beom Jeon (23) reported that patients with a reduction of midline shift following decompressive hemicraniectomy for MMCAI were more likely to be alive at 30-day poststroke than those without. In this study, the improvement in midline shift was a strong predictor for a good prognosis (*t* = 4.214, *p* < 0.01) (Table 1, Figure 1C) in the univariate analysis. And in multivariable analysis, the improvement of the midline shift was a protector against the death of patients at 30 days after DC (OR = 0.764, 95% CI 0.59–0.988), which meant that if the midline shift returned, the surgical patient was more likely to be alive at 30 days after DC. We used the ROC analysis to obtain a cutoff value of midline shift improvement to predict the outcome of the DC patients. The midline shift improvement of more than 0.83 cm was the most sensible predictor for survival with an AUC of 0.844 (Figure 3).

## Conclusion

In this study, we showed that increasing age, right hemisphere location, hypertension, and pupillary dilation were predictors for the poor outcome of death for MMCAI patients after DC operation; however, midline shift improvement was a protective predictor against death. Its predictive value peaked at 0.83 cm. Further prospective randomized trials with more enrolled patients may prove useful in defining the indication of craniectomy after stroke.

## Data Availability Statement

The original contributions presented in the study are included in the article/supplementary material, further inquiries can be directed to the corresponding author/s.

## Ethics Statement

Written informed consent was obtained from the individual(s) for the publication of any potentially identifiable images or data included in this article.

## Author Contributions

All authors listed have made a substantial, direct and intellectual contribution to the work, and approved it for publication.

## Conflict of Interest

The authors declare that the research was conducted in the absence of any commercial or financial relationships that could be construed as a potential conflict of interest.
